# Extension of the Consolidated Criteria for Reporting Qualitative Research Guideline to Large Language Models (COREQ+LLM): Protocol for a Multiphase Study

**DOI:** 10.2196/78682

**Published:** 2025-09-24

**Authors:** Leonard Fehring, Julian Frings, Paul Rust, Christian Kempny, Petra A Thürmann, Sven Meister

**Affiliations:** 1 Health Care Informatics Faculty of Health, School of Medicine Witten/Herdecke University Witten Germany; 2 Department of Gastroenterology Helios Universitätsklinikum Wuppertal Wuppertal Germany; 3 Health Services Research Faculty of Health, School of Medicine Witten/Herdecke University Witten Germany; 4 Hamm-Lippstadt University of Applied Sciences Hamm Germany; 5 Chair of Clinical Pharmacology Faculty of Health, School of Medicine Witten/Herdecke University Witten Germany; 6 Department Healthcare Fraunhofer Institute for Software and Systems Engineering Dortmund Germany

**Keywords:** qualitative research, large language models, LLMs, Consolidated Criteria for Reporting Qualitative Research, COREQ, reporting guideline, artificial intelligence, AI

## Abstract

**Background:**

Qualitative research provides essential insights into human behaviors, perceptions, and experiences in health sciences. The COREQ (Consolidated Criteria for Reporting Qualitative Research) checklist, published in 2007 and endorsed by the Enhancing the Quality and Transparency of Health Research Network, advanced transparency of qualitative research reporting. However, the recent integration of large language models (LLMs) into qualitative research introduces novel opportunities and methodological challenges that existing guidelines do not address. LLMs are increasingly applied to research design as well as processing, analysis, interpretation, and even direct interaction (“conversing”) with qualitative data. However, their probabilistic nature, dependence on underlying training data, and susceptibility to hallucinations necessitate dedicated reporting to ensure transparency, reproducibility, and methodological validity.

**Objective:**

This protocol outlines the methodological development process of COREQ+LLM, an extension to the COREQ checklist, to support transparent reporting of LLM use in qualitative research. The three main objectives are to (1) identify and categorize current applications of LLMs used as qualitative research tools, (2) assess how LLM use in qualitative studies in health care is reported in published studies, and (3) develop and refine reporting items for COREQ+LLM through a structured consensus process among international experts.

**Methods:**

Following the Enhancing the Quality and Transparency of Health Research Network guidance for reporting guideline development, this study comprises 4 main phases. Phase 1 is a systematic scoping review of peer-reviewed literature from January 2020 to April 2025, examining the use and reporting of LLMs in qualitative research. The scoping review protocol was registered with the Open Science Framework on June 6, 2025, and will adhere to the PRISMA-ScR (Preferred Reporting Items for Systematic Reviews and Meta-Analyses Extension for Scoping Reviews) guidelines. Phase 2 will use a Delphi process to reach consensus on candidate items for inclusion in the COREQ+LLM checklist among an interdisciplinary international panel of experts. Phase 3 includes pilot testing, and phase 4 involves publication and dissemination.

**Results:**

As of September 2025, the steering committee has been established, and the initial search strategy for the scoping review has identified 5049 records, with 4201 (83.20%) remaining after duplicate removal. Title and abstract screening is underway and will inform the initial draft of candidate checklist items. The COREQ+LLM extension is scheduled for completion by December 2025.

**Conclusions:**

The integration of LLMs in qualitative research requires dedicated reporting guidelines to ensure methodological rigor, transparency, and interpretability. COREQ+LLM will address current reporting gaps by offering specific guidance for documenting LLM integration in qualitative research workflows. The checklist will assist researchers in transparently documenting LLM use, support reviewers and editors in evaluating methodological quality, and foster trust in LLM-supported qualitative research. By December 2025, COREQ+LLM will provide a rigorously developed tool to enhance the transparency, validity, and reproducibility of LLM-supported qualitative studies.

**International Registered Report Identifier (IRRID):**

DERR1-10.2196/78682

## Introduction

### Background

Qualitative research serves a foundational role in health sciences by providing deep, contextualized insights into human behaviors, perceptions, and lived experiences [1-3]. Unlike quantitative approaches, qualitative studies use non-numerical methods, enabling detailed exploration and generating novel perspectives within health care [1,4]. Qualitative research encompasses diverse methodologies, predominantly involving interviews, focus groups, participant observation, and analysis of documents or artifacts, supported by analytic techniques, such as coding, thematic analysis, and content analysis [5,6].

Ensuring rigor and transparency in qualitative research requires standardized reporting guidelines. Since its publication in 2007, the COREQ (Consolidated Criteria for Reporting Qualitative Research) checklist has become widely accepted, with endorsements from organizations such as the Enhancing the Quality and Transparency of Health Research (EQUATOR) Network, and has been cited more than 30,000 times [7]. COREQ supports methodological transparency and clarity and, consequently, the interpretability and trustworthiness of qualitative findings.

However, qualitative research methodologies are evolving rapidly due to the accelerated integration of large language models (LLMs), a type of artificial intelligence (AI). These technologies introduce both unprecedented opportunities and novel methodological challenges beyond the scope of the original COREQ guidelines. LLMs, trained on extensive text corpora, generate coherent, contextually relevant text in response to user inputs (prompts) [8]. Their capacity to analyze, interpret, and generate humanlike language underpins their increasing use across qualitative research and practical applications.

The integration of LLMs into qualitative research workflows is expanding rapidly. Researchers use these models at multiple stages, including formulating research questions; designing interview protocols; supporting data processing tasks, such as transcription and translation; aiding analytic processes, such as coding and theme identification; drafting manuscript sections; and facilitating interactive dialogue with qualitative datasets [9-19]. This adoption is largely motivated by the perceived benefits offered by LLMs [20]. Notably, LLMs can enhance the speed and efficiency of data analysis by rapidly processing extensive textual datasets, enabling researchers to undertake more expansive and expedited investigations. Beyond efficiency, LLMs provide novel analytic perspectives by identifying latent semantic patterns, proposing new thematic frameworks, and distilling complex narrative content. These capabilities complement human interpretive efforts and have potential implications for reducing interpretive biases. Consequently, leading qualitative analysis software packages, including ATLAS.ti and MAXQDA, now integrate LLM functionalities [21,22].

Despite these advantages, the integration of LLMs into qualitative research introduces several methodological risks and necessitates rigorous reporting standards. Unlike conventional software tools that often rely on deterministic algorithms, LLMs function probabilistically. While adept at identifying and reproducing language patterns from training data, their semantic comprehension, particularly regarding nuanced contexts and subtexts, remains uncertain and under active scientific investigation [23]. Furthermore, because LLMs are trained on large-scale datasets that reflect statistical regularities within human language, they are susceptible to reinforcing existing biases, perpetuating dominant cultural narratives, and systematically underrepresenting marginalized perspectives [24,25]. The internal mechanisms guiding their outputs are often opaque (“black box” phenomenon), which poses significant challenges for interpretability and explainability. In addition, LLMs are prone to producing outputs that are syntactically plausible yet factually inaccurate, a behavior known as “hallucination” [26]. In the context of qualitative research, such inaccuracies may be especially difficult to detect due to interpretive depth and narrative complexity. These factors collectively introduce critical challenges to reproducibility, validity, and overall trustworthiness of qualitative research facilitated by LLMs.

Reflecting these concerns, the qualitative research community is actively debating the appropriate role and regulation of LLMs. Schroeder et al [27], after interviewing 20 qualitative researchers, highlighted an “urgent lack of norms” governing model use and advocated for tool-supported transparency. Zhang et al [28] further argued that enhancing transparency and deepening researchers’ understanding of LLM capabilities can improve their effective use. Similarly, in a workshop involving 40 scholars, Quéré et al [29] observed that LLMs have already been integrated into many researchers’ workflows; however, they emphasized the need for defined standards to assess validity and rigor that, at present, remain undefined.

The existing COREQ checklist offers insufficient guidance for navigating these complexities. Its only applicable item (item 27: “What software, if applicable, was used to manage the data?”) inadequately captures essential methodological details needed to assess the implications of LLM use. Merely naming software or models used is insufficient; understanding precise use contexts, limitations, and verification strategies is crucial for evaluating research quality.

Existing reporting guidelines for AI, such as TRIPOD-LLM (Transparent Reporting of a Multivariable Prediction Model for Individual Prognosis or Diagnosis–Large Language Model), CONSORT-AI (Consolidated Standards of Reporting Trials-Artificial Intelligence), and SPIRIT-AI (Standard Protocol Items: Recommendations for Interventional Trials-Artificial Intelligence), primarily address areas that are not specific to qualitative research. Instead, these guidelines focus on topics such as the development or evaluation of LLMs, randomized controlled trials involving AI, and clinical trial protocols evaluating interventions with AI, respectively [30-32].

Recognizing this critical methodological gap, we are developing COREQ+LLM, a rigorous extension of the established COREQ checklist. COREQ+LLM will introduce specific reporting items essential for transparently describing and critically evaluating qualitative research incorporating LLMs. This extension aims to capture detailed methodological information, enabling a thorough appraisal of rigor, trustworthiness, and reproducibility, thereby maintaining the foundational integrity of qualitative research in the era of AI.

### Objectives

The overall objective of this study is to extend the existing COREQ checklist to support transparent and responsible reporting of LLMs in qualitative research. To achieve this, we aim to (1) identify and categorize current applications of LLMs used as qualitative research tools (eg, conducting interviews, analyzing focus group discussions, and extracting themes from textual data; refer to Inclusion and Exclusion criteria section for more details), (2) assess how LLM use as a qualitative research tool is reported in peer-reviewed empirical studies, and (3) develop and refine reporting items for COREQ+LLM through a structured consensus process among international experts.

Compared with earlier natural language processing (NLP) approaches, such as statistical n-gram models or task-specific neural networks, LLMs function as general-purpose problem-solvers [33]. Their breadth of capability arises from massively scaling model parameters, training data, and compute used for training [34]. While traditional NLP pipelines were engineered for narrowly defined tasks, such as feature extraction, LLMs can generalize across an extensive array of linguistic tasks, which they were never explicitly trained to perform. Therefore, focusing the COREQ extension on LLMs is warranted on both technical and pragmatic grounds. From a technical perspective, LLMs possess generative capacities that create entirely new epistemic objects (eg, full paragraphs, code blocks, or analytic summaries) rather than merely classifying or extracting text. These capabilities introduce LLM-specific risks that are intrinsic to the model architecture, notably hallucinations and nondeterministic outputs [35]. From a pragmatic standpoint, expanding our checklist to encompass other AI modalities, such as computer vision, reinforcement learning, symbolic AI, or rule-based NLP, would render the resulting framework overly complex and risk impeding consensus among researchers. Given that qualitative research fundamentally engages with linguistic artifacts, such as interviews and documents, maintaining a language-first focus ensures that our guidance remains aligned with the empirical substrate of qualitative inquiry while avoiding an unwieldy, modality-agnostic approach.

At the same time, the integration of LLMs into qualitative research represents part of a broader trend toward the adoption of AI in qualitative research. By focusing on LLMs, COREQ+LLM provides a timely and targeted response to the most prominent and methodologically disruptive form of AI currently impacting qualitative inquiry. This extension can serve as a foundational step toward broader guidance on AI-supported qualitative research while ensuring actionable and consensus-driven reporting standards for the most immediate use cases. For these reasons, we propose the guideline extension to be titled “COREQ+LLM” rather than “COREQ+AI.”

## Methods

### Overview

The development of the COREQ+LLM extension adheres to internationally recognized methodologies for creating health research reporting guidelines, primarily drawing upon the guideline development approach provided by the EQUATOR Network [36]. This approach aligns with precedents set by other AI-focused reporting guideline extensions, such as TRIPOD-LLM, CONSORT-AI, and SPIRIT-AI [30-32]. Recognizing the complexity and rapid evolution of LLM technologies, we use a rigorous, multiphase methodology, comprising a scoping review (phase 1) and a Delphi consensus process (phase 2). Given the rapid evolution of the use of LLMs in qualitative health research and the lack of standardization, a Delphi method allows for structured expert deliberation while integrating emergent, interdisciplinary knowledge [37]. Table 1 summarizes the research process.

**Table 1:** Summary of the process for development of the COREQ+LLM (Consolidated Criteria for Reporting Qualitative Research and Large Language Models) extension.

Phase and description		Goals	Timeline
**Phase 0: preparation**			
	Establish a steering committee consisting of clinicians, methodologists, and researchers with vast experience in qualitative data analysis and LLMs to oversee guideline development	Governance and oversight set	April 2025 to May 2025
	Register extension with the EQUATOR^a^ network as a guideline under development; publish the study protocol	Duplication of efforts prevented	June 2025
**Phase 1: scoping review**			
	Conduct a scoping review in accordance with PRISMA-ScR^b^ to identify health care journal articles using LLMs for qualitative research to identify potential candidate items for checklist extension and current best practices	List of potential items created (first set)	March 2025 to June 2025
**Phase 2: Delphi rounds**			
	Conduct an online Delphi survey in accordance with DELPHISTAR^c^ among multispecialty experts to assess the first set of extension candidates and collect suggestions for additional items; conduct additional Delphi rounds, as needed; ratings on a 5-point Likert scale, with consensus defined as more than 75% agreement	First set of items assessed and additional items added	July 2025 to September 2025
	Hold a consensus meeting to discuss disagreements and reach a final consensus (>75% agreement) on the inclusion or exclusion of items	Consensus achieved and draft of final COREQ+LLM checklist created	September 2025
**Phase 3: pilot testing and finalization**			
	Pilot-test the COREQ+LLM checklist with researchers conducting LLM-supported qualitative health research and revise COREQ+LLM accordingly	COREQ+LLM pilot-tested and finalized	October 2025 to November 2025
**Phase 4: dissemination**			
	Publish COREQ+LLM in a journal and on the EQUATOR website, including an explanation and elaboration document	COREQ+LLM published and disseminated	December 2025

^a^EQUATOR: Enhancing the Quality and Transparency of Health Research.

^b^PRISMA-ScR: Preferred Reporting Items for Systematic Reviews and Meta-Analyses Extension for Scoping Reviews.

^c^DELPHISTAR: Delphi studies in social and health sciences – recommendations for an interdisciplinary standardized reporting.

### Phase 0: Preparation

This study will be overseen by a 6-member steering committee comprising AI researchers, clinicians, ethicists, experts in qualitative research methodology, and representatives for good scientific practice from Witten/Herdecke University. The protocol will be registered with the EQUATOR Network as a reporting guideline under development. The scoping review protocol was registered with the Open Science Framework on June 6, 2025 [38], and adheres to the PRISMA-ScR (Preferred Reporting Items for Systematic Reviews and Meta-Analyses Extension for Scoping Reviews) guidelines [39].

### Phase 1: Scoping Review

#### Review Questions

The scoping review addresses 2 primary questions aligned with our objectives: (1) What are the current applications of LLMs as qualitative research tools? (2) What specific reporting practices are used in studies that use LLMs for conducting qualitative research?

#### Inclusion and Exclusion Criteria

We include peer-reviewed, discipline-agnostic articles published between January 1, 2020, and May 27, 2025, written in English, which use at least 1 qualitative method supported by an LLM for tasks beyond manuscript writing improvement. Conference presentations and abstracts are excluded.

LLM applications will be defined as instances where an LLM is used as a qualitative research tool, that is, to support or automate tasks traditionally conducted in qualitative research, such as interviewing, transcription, thematic coding, content analysis, or interpretive synthesis. We will exclude studies in which LLMs are only the subject of evaluation (eg, studies assessing model performance or accuracy) or those focused solely on perceptions, attitudes, or acceptance of LLMs. Only studies in which the LLM actively contributes to the qualitative research process will be included.

The restriction to English-language publications is based primarily on the dominance of English as the main language of publication in LLM and qualitative health research, where most of the methodological developments and applications are disseminated. To assess potential bias, we conducted a targeted search using equivalent German-language terms as a model check and found no eligible studies, suggesting that the likelihood of excluding relevant non-English literature is low.

The 5-year time frame is justified by the recent emergence and rapid evolution of LLMs. While ChatGPT (OpenAI), the most widely used generative LLM, was released in November 2022, we aim to capture not only early research involving ChatGPT but also earlier applications of generative LLMs, including those based on preceding models. Extending the time frame beyond 5 years is not appropriate, as the use of LLMs to support qualitative research did not exist before this period.

If the initial search strategy yields an insufficient number of peer-reviewed articles (fewer than 5 articles), we will supplement the review with preprints sourced from Scopus Preprint.

#### Search Strategy and Study Selection

We developed a comprehensive search strategy across the databases APA PsycINFO, PubMed, Business Source Premier, Scopus, and CINAHL, informed by an initial literature snowballing process. Keywords, synonyms, and Medical Subject Headings terms were used (see Multimedia Appendix 1). Duplicates will be automatically removed based on digital object identifiers. Three reviewers will independently screen titles and abstracts to determine eligibility, with inclusion requiring agreement by at least 2 reviewers. Data extraction will be validated by a fourth reviewer.

We will document the full study selection process, including the number of records identified, screened, assessed for eligibility, and included in the final synthesis. Reasons for exclusion at the full-text stage will also be recorded. This process will be transparently presented using a PRISMA-ScR flow diagram.

#### Data Analysis and Synthesis

The extracted data will include study metadata, study characteristics (eg, study aim, study design, and type of qualitative method used), the details of the LLM application (eg, model configuration, prompting approaches, and validation strategies), and the level of reporting transparency (eg, presence of statements regarding ethical considerations, LLM-specific biases, and role of LLMs’ interpretive analysis). Three reviewers will independently and in parallel complete an initial round of data extraction using a specifically developed data charting form. This will be followed by a joint discussion to determine whether the form requires adaptation. In case of persistent disagreements, a fourth reviewer will arbitrate. In the second round, data will be re-extracted using the updated form to ensure consistency. Finally, all reviewers will examine the completed form, resolving any disagreements through consensus to ensure uniformity in the data charting process.

Extracted data will include study type (eg, interview, thematic analysis, and ethnography), publication year, discipline, country of origin, and detailed reporting of LLM use (eg, model version, parameter settings, use case, and prompting). The synthesis will involve frequency mapping of selected variables (eg, study metadata, characteristics, and LLM application types) and narrative summaries for others (eg, reporting transparency). Together, these analyses will provide an integrated synthesis of LLM application and reporting characteristics, informing a preliminary candidate item list for the guideline extension. Stakeholder consultation with the steering committee may be conducted to validate the candidate list before the Delphi consensus process.

### Phase 2: Delphi Consensus Process

#### Overview

The goal of the Delphi consensus process is to systematically elicit expert judgment to validate, refine, and add items and ultimately achieve expert consensus on the content of COREQ+LLM. The Delphi process will evaluate, refine, and finalize candidate reporting items derived from the phase 1 scoping review.

#### Design

We will conduct a Delphi study to meet the pressing need for timely reporting guidelines in qualitative research involving LLMs. The Delphi study will be guided by the Delphi studies in social and health sciences–recommendations for an interdisciplinary standardized reporting framework recommended by the EQUATOR network for Delphi design and reporting [37,40]. Experts will independently and anonymously evaluate each candidate item and suggest additions or changes until group consensus on the COREQ+LLM reporting items is achieved. These experts are the participants of the Delphi study. Participants will be provided with structured aggregated results to help reconcile individual differences and achieve group consensus. The list of candidate items for COREQ+LLM will be collated by the steering committee based on the results of the scoping review. Items will be pilot-tested for clarity, relevance, and comprehensibility before round 1. This pretest aims to identify ambiguous wording, improve clarity, and ensure the relevance of each candidate item before round 1 [40,41]. The Delphi surveys will be hosted electronically on LimeSurvey. Information sheets and consent forms will be embedded within the platform. Delphi participants can choose to remain anonymous. Contact information is collected to provide results, invite participants to the subsequent rounds and the consensus meeting, and provide the option to be named in the acknowledgments. Contact information will be collected separately and stored independently from survey responses. Data processing will follow the General Data Protection Regulation–compliant procedures, including pseudonymization, voluntary consent, and restricted access to personal data. No identifying information will be linked to Delphi responses, and all data will be used solely for scientific purposes.

The results of the first round will be calculated as described subsequently. The steering committee will determine when to proceed to the online consensus meeting. Divergent views will be retained and analyzed by the steering committee to inform item refinement. This Delphi study adopts a constructivist epistemological stance, recognizing that consensus reflects situated expert perspectives rather than objective truth [41].

#### Recruitment Process and Expert Panel Selection

Delphi study participants will be identified by the steering committee via publications, professional networks of the steering committee, and snowball sampling from initial recruits. They will be vetted by the steering committee based on criteria such as publication history, methodological experience, and recognized contributions to qualitative health research. The Delphi study participants should represent geographic and disciplinary diversity, including key stakeholder groups, for example, researchers (particularly those conducting qualitative research in health care), health care professionals, journal editors, funders, policymakers, health care regulators, and patient advocate groups. Potential participants will receive personalized invitations via email as well as 2 reminders. The email will describe the objectives, process, and timeline of the Delphi consensus process to develop COREQ+LLM. We aim to invite at least 100 participants. In the event of low initial response or substantially attrition between rounds, we will issue additional reminders and consider extending the response window or inviting additional experts from the same stakeholder groups to maintain adequate participation and panel diversity. Participants may be acknowledged by name in project publications, if desired. Each Delphi round will remain open for 3 weeks, with reminders sent after 1 and 2 weeks.

#### Online Delphi Rounds

Participants will be asked to consider the following guiding principles when assessing the candidate items for exclusion or inclusion in COREQ+LLM: (1) items should be broadly applicable across qualitative health care studies using LLMs while allowing for assessment of contextual relevance; (2) items should promote transparent reporting of how LLMs were integrated into the qualitative research process, enabling understanding and potential reproducibility; (3) items should support appraisal of methodological rigor and potential biases introduced through LLM use; (4) the overall set should reflect the minimum information needed to responsibly report LLM-supported qualitative research; and (5) items should encourage ethical and epistemic accountability, including reflection on the implications of using LLMs in health research.

Participants will rate each candidate reporting item on a 5-point Likert scale as follows: “5—essential for inclusion,” “4—desirable for inclusion,” “3—neutral or unsure,” “2—rather not include,” and “1—definitely exclude” following a similar scale used in the development of TRIPOD+AI [42]. Consensus will be defined as more than 75% of participants rating an item as “desirable for inclusion” or “essential for inclusion” following internationally established consensus thresholds [43,44]. Items with 50% agreement or less will be excluded. Items with more than 75% agreement will be included in COREQ+LLM. Items with majority agreement but no consensus (>50% and ≤75%) will be merged or rephrased to be presented to the expert panel for re-evaluation in the next round. In addition, participants in round 1 can provide free-text suggestions for adding, merging, or altering items. Free-text responses will be thematically analyzed by 2 researchers and carried to round 2. Responses will be aggregated across all participants and summarized using descriptive statistics (eg, frequencies and proportions for each rating category), alongside a qualitative synthesis of the free-text comments. Participants will receive aggregated quantitative results and an anonymized synthesis of free-text responses from the previous round to inform their reassessment. We anticipate conducting at least 2 Delphi rounds. If substantial disagreement persists or many items fall within the intermediate range, a third online Delphi round will be initiated to further refine the list of candidate items.

#### Consensus Meeting

An online consensus meeting will be conducted to re-evaluate items from the online Delphi rounds that received the most support (>50%) but did not reach the predefined consensus threshold (>75% agreement). The primary goal of this meeting is to finalize the set of reporting items to be included in COREQ+LLM. The meeting will include a subgroup of up to 20 participants from the previous Delphi rounds, selected by the steering committee to ensure broad stakeholder representation. Before the meeting, participants of the consensus meeting will receive a detailed agenda, along with the results of the scoping review and a summary of the previous round’s findings, compiled by the steering committee. During the meeting, steering committee members will facilitate a structured discussion of each outstanding item, and participants will vote on each item using a live polling tool. To minimize bias, the live polling will be anonymous, and steering committee members will remain nonvoting. Items reaching more than 75% agreement for inclusion will be considered as having achieved consensus and included in the final COREQ+LLM checklist. During the consensus meeting, items lacking consensus will be discussed with reference to dissenting viewpoints. If consensus remains unattainable, these items may be documented as contested, along with the rationale, in the final checklist or accompanying elaboration documents. The meeting will conclude with agreement on a draft version of COREQ+LLM.

### Phase 3: Pilot Testing

The steering committee will oversee the development of the COREQ+LLM explanation and elaboration document, with draft versions circulated to consensus meeting participants for review and feedback. To enhance clarity and usability, pilot testing will be conducted with 3 to 5 researchers, including PhD students, who are currently conducting qualitative research involving LLMs as a research tool. Feedback will be used to identify any items that are unclear or difficult to interpret. Participants will apply the draft checklist to a relevant study or their own work and provide structured feedback on item clarity, relevance, and ease of use. On the basis of this input, the steering committee may revise the checklist (adding, modifying, or removing items) as necessary to ensure the final version is both comprehensive and practical.

### Phase 4: Dissemination

We plan to disseminate COREQ+LLM widely by publishing it in a peer-reviewed journal, presenting it at major academic and clinical conferences, and making it freely accessible to the global research community through the EQUATOR Network website [45].

### Ethical Considerations

The ethics committee of Witten/Herdecke University raised no objection regarding ethical concerns (S-165/2025). Each participant will be asked to provide informed consent prior to their participation in the Delphi study. No financial incentives will be offered to participants.

## Results

As of May 2025, the 6-person steering committee to oversee COREQ+LLM development has been established (the authors of this protocol). The search strategy identified 5049 records; after duplicate removal, 4201 (83.20%) studies progressed to title and abstract screening. The title and abstract screening process is currently ongoing and will inform the initial draft of candidate checklist items. We anticipate completing the scoping review and Delphi process, along with publication of COREQ+LLM results, by December 2025.

## Discussion

### Anticipated Findings

The integration of LLMs into qualitative research signifies a substantial shift in methodologies for data collection, analysis, and interpretation. Without specialized reporting guidance, there is a risk to the quality, transparency, and trustworthiness of research involving LLMs. We anticipate the scoping review will reveal variability and gaps in reporting practices concerning the use of LLMs, reinforcing the need for structured guidelines. Given these challenges, we expect great stakeholder interest in the Delphi process, as the absence of clear reporting standards constitutes a considerable burden. COREQ+LLM aims to bridge this critical gap by offering a comprehensive, consensus-driven framework for transparently reporting the use of LLMs in qualitative research.

### Anticipated Impact

The COREQ+LLM guideline is designed to support diverse stakeholders. Researchers will gain structured guidance for transparently documenting LLM use; peer reviewers and journal editors will have a robust tool to evaluate the completeness and methodological rigor of submissions; clinicians, policymakers, patients, and other researchers will benefit from enhanced clarity and confidence when interpreting LLM-assisted qualitative findings. Ultimately, COREQ+LLM seeks to improve the credibility, validity, and practical relevance of qualitative research using advanced LLM-supported methodologies.

### Comparison to Prior Work

To the best of our knowledge and based on a review of the “reporting guidelines under development for other study designs” section of the EQUATOR Network, there are no reporting guidelines that specifically address the use of LLMs in qualitative research [46]. AI-focused extensions of reporting guidelines exist (eg, CONSORT-AI, TRIPOD-LLM, and SPIRIT-AI); however, they do not address the specific methodological implications of using LLMs in qualitative inquiry. Existing guidelines, such as COREQ, contain only minimal references to software use and do not account for the probabilistic, generative, and opaque characteristics of LLMs. COREQ+LLM addresses this gap by focusing on the use of LLMs as qualitative research tools rather than their evaluation or perception.

### Strengths and Limitations

A major strength of this protocol is its adherence to EQUATOR and DELPHISTAR guidance, ensuring methodological rigor in both the scoping review (registered with the Open Science Framework [38]) and the Delphi consensus process. One potential limitation is that expert participation in Delphi studies can vary; therefore, strategies to mitigate low response rates have been included. Furthermore, the scoping review will be restricted to English-language publications, and although the steering committee will be purposefully composed to reflect diverse geographic regions and disciplines, it may not encompass all perspectives. These factors may influence the generalizability of the final checklist.

### Future Directions

Upon completion, COREQ+LLM may serve as a foundation for broader extensions addressing other AI modalities in qualitative research (eg, computer vision). It may also stimulate further methodological research into the integration of LLMs and hybrid human-AI analytic workflows in qualitative research studies.

### Dissemination Plan

The results from this study, including the final COREQ+LLM checklist and explanation and elaboration document, will be published in a peer-reviewed journal and submitted to the EQUATOR Network for public use (refer to phase 4: Dissemination section). Moreover, we will plan to present findings at relevant academic and clinical conferences to promote awareness and uptake across the qualitative health research community.

### Conclusions

COREQ+LLM responds to the growing use of LLMs as a qualitative research tool by providing structured, consensus-based guidance for transparent reporting. This protocol outlines a rigorous, multiphase development process that combines systematic evidence synthesis with expert consensus through a Delphi process. The resulting guideline aims to enhance trust, reproducibility, and methodological transparency in LLM-assisted qualitative research.

## Appendix

Multimedia Appendix 1 Overview of the search strategy.

## Abbreviations

AI: artificial intelligence
CONSORT-AI: Consolidated Standards of Reporting Trials-Artificial Intelligence
COREQ: Consolidated Criteria for Reporting Qualitative Research
EQUATOR: Enhancing the Quality and Transparency of Health Research
LLM: large language model
NLP: natural language processing
PRISMA-ScR: Preferred Reporting Items for Systematic Reviews and Meta-Analyses Extension for Scoping Reviews
SPIRIT-AI: Standard Protocol Items: Recommendations for Interventional Trials-Artificial Intelligence
TRIPOD-LLM: Transparent Reporting of a Multivariable Prediction Model for Individual Prognosis or Diagnosis-Large Language Model
