# Supplementary material for: Extension of the Consolidated Criteria for Reporting Qualitative Research Guideline to Large Language Models (COREQ+LLM): Protocol for a Multiphase Study
Source: JMIR Res Protoc. 2025 Sep 24;14:e78682. doi: 10.2196/78682 (PMC12508663; doi:10.2196/78682)
Supplement: Multimedia Appendix 1 [file resprot_v14i1e78682_app1.pdf]

Table 1. Search strategy used in the scoping review.

Database	Search strategy	Anticipated articles, n
Scopus	(TITLE-ABS-KEY({large language model} OR {generative AI} OR {foundation model} OR {transformer model} OR {prompt engineering} OR {automated text analysis} OR {conversational AI}) AND TITLE-ABS-KEY({thematic analysis} OR {qualitative research} OR {content analysis} OR {discourse analysis} OR {interpretative phenomenological analysis} OR {participant observation} OR {reflexive thematic analysis} OR {autoethnography} OR {member checking} OR {data saturation} OR {inductive analysis} OR {deductive analysis} OR {focus groups} OR {interviews} OR {narratives} OR {observation})) AND PUBYEAR > 2019 AND PUBYEAR < 2026 AND (LIMIT-TO (SRCTYPE,“j”)) AND (LIMIT-TO (DOCTYPE,“ar”)) AND (LIMIT-TO (SUBJAREA,“MEDI”) OR LIMIT-TO (SUBJAREA,“SOCI”) OR LIMIT-TO (SUBJAREA,“PSYC”) OR LIMIT-TO (SUBJAREA,“HEAL”) OR LIMIT-TO (SUBJAREA,“NURS”)) AND (LIMIT-TO (LANGUAGE,“English”))	636
PubMed	(“Large Language Model*”[Title/Abstract] OR Generative AI[Title/Abstract] OR GPT[Title/Abstract] OR “Artificial Intelligence”[Title/Abstract] OR	2111

	AI*[Title/Abstract] OR "Machine Learning"[Title/Abstract] OR ML[Title/Abstract] OR "Natural Language Processing"[Title/Abstract] OR* NLP*[Title/Abstract] OR ChatGPT[Title/Abstract] OR* LLM*[Title/Abstract] OR "Deep Learning"[Title/Abstract] OR "Transformer Model"[Title/Abstract] OR "Neural Network*"[Title/Abstract] OR "Text Mining"[Title/Abstract] OR "Automated Text Analysis"[Title/Abstract] OR "Conversational* AI"*[Title/Abstract] OR "Generative Model*"[Title/Abstract] OR "Foundation Model*"[Title/Abstract] OR "Prompt Engineering"[Title/Abstract]) AND (("Thematic Analysis"[Title/Abstract] OR "Content Analysis"[Title/Abstract] OR "Qualitative Research"[Title/Abstract] OR "Interpretative Phenomenological Analysis"[Title/Abstract] OR "Discourse Analysis"[Title/Abstract] OR "Ethnography"[Title/Abstract] OR "Participant Observation"[Title/Abstract] OR "Reflexive Thematic Analysis"[Title/Abstract] OR "Autoethnography"[Title/Abstract] OR "Member Checking"[Title/Abstract] OR "Data Saturation"[Title/Abstract] OR "Inductive Analysis"[Title/Abstract] OR "Deductive Analysis"[Title/Abstract]) OR ("Empirical Research"[Mesh] OR "Focus Groups"[Mesh] OR "Interviews as Topic"[Mesh] OR "Narration"[Mesh] OR "Observation"[Mesh]))*	

CINAHL	((TI "Large Language Model*" OR AB "Large Language Model*" OR TI "Generative AI" OR AB "Generative AI" OR TI GPT OR AB GPT OR TI "Artificial Intelligence" OR AB "Artificial Intelligence" OR TI AI OR AB AI OR TI "Machine Learning" OR AB "Machine Learning" OR TI ML OR AB ML OR TI "Natural Language Processing" OR AB "Natural Language Processing" OR TI NLP OR AB NLP OR TI ChatGPT OR AB ChatGPT OR TI LLM OR AB LLM OR TI "Deep Learning" OR AB "Deep Learning" OR TI "Transformer Model" OR AB "Transformer Model" OR TI "Neural Network*" OR AB "Neural Network*" OR TI "Text Mining" OR AB "Text Mining" OR TI "Automated Text Analysis" OR AB "Automated Text Analysis" OR TI "Conversational AI" OR AB "Conversational AI" OR TI "Generative Model*" OR AB "Generative Model*" OR TI "Foundation Model*" OR AB "Foundation Model*" OR TI "Prompt Engineering" OR AB "Prompt Engineering")) AND ((TI "Thematic Analysis" OR AB "Thematic Analysis" OR TI "Content Analysis" OR AB "Content Analysis" OR TI "Qualitative Research" OR AB "Qualitative Research" OR TI "Interpretative Phenomenological Analysis" OR AB "Interpretative Phenomenological Analysis" OR TI "Discourse Analysis" OR AB "Discourse Analysis" OR TI Ethnography OR AB Ethnography OR TI "Participant Observation"	726

	OR AB *"Participant Observation" OR* TI *"Reflexive Thematic Analysis" OR* AB *"Reflexive Thematic Analysis" OR* TI *Autoethnography OR* AB *Autoethnography OR* TI *"Member Checking" OR* AB *"Member Checking" OR* TI *"Data Saturation" OR* AB *"Data Saturation" OR* TI *"Inductive Analysis" OR* AB *"Inductive Analysis" OR* TI *"Deductive Analysis" OR* AB *"Deductive Analysis" OR* DE *"Empirical Research" OR* DE *"Focus Groups" OR* DE *"Interviews" OR* DE *"Narratives" OR* DE *"Observation")*	
PsycINFO	*((*TI *"Large Language Model*" OR AB "Large Language Model*" OR TI "Generative* AI*" OR AB "Generative* AI*" OR TI* GPT *OR* AB GPT *OR* TI *"Artificial Intelligence" OR* AB *"Artificial Intelligence" OR* TI AI *OR* AB AI *OR* TI *"Machine Learning" OR* AB *"Machine Learning" OR* TI ML *OR* AB ML *OR* TI *"Natural Language Processing" OR AB "Natural Language Processing" OR TI* NLP *OR* AB NLP *OR* TI *ChatGPT OR* AB *ChatGPT OR* TI LLM *OR* AB LLM *OR* TI *"Deep Learning" OR AB "Deep Learning" OR TI "Transformer Model" OR AB "Transformer Model" OR TI "Neural Network*" OR AB "Neural Network*" OR TI "Text Mining" OR AB "Text Mining" OR TI "Automated Text Analysis" OR AB "Automated Text Analysis" OR TI "Conversational* AI*" OR AB "Conversational* AI*" OR TI "Generative Model*" OR AB "Generative Model*" OR TI "Foundation*	678

	Model" OR AB "Foundation Model*" OR TI "Prompt Engineering" OR AB "Prompt Engineering")) AND ((TI "Thematic Analysis" OR* AB *"Thematic Analysis" OR* TI *"Content Analysis" OR* AB *"Content Analysis" OR* TI *"Qualitative Research" OR* AB *"Qualitative Research" OR* TI *"Interpretative Phenomenological Analysis" OR* AB *"Interpretative Phenomenological Analysis" OR* TI *"Discourse Analysis" OR* AB *"Discourse Analysis" OR* TI *Ethnography OR* AB *Ethnography OR* TI *"Participant Observation" OR* AB *"Participant Observation" OR* TI *"Reflexive Thematic Analysis" OR* AB *"Reflexive Thematic Analysis" OR* TI *Autoethnography OR* AB *Autoethnography OR* TI *"Member Checking" OR* AB *"Member Checking" OR* TI *"Data Saturation" OR* AB *"Data Saturation" OR* TI *"Inductive Analysis" OR* AB *"Inductive Analysis" OR* TI *"Deductive Analysis" OR* AB *"Deductive Analysis" OR* DE *"Empirical Research" OR* DE *"Focus Groups" OR* DE *"Interviews" OR* DE *"Narratives" OR* DE *"Observation")*	
BSP	*((*TI *"Large Language Model*" OR AB "Large Language Model*" OR TI "Generative* AI*" OR AB "Generative* AI*" OR TI* GPT *OR* AB GPT *OR* TI *"Artificial Intelligence" OR* AB *"Artificial Intelligence" OR* TI AI *OR* AB AI *OR* TI *"Machine Learning" OR* AB *"Machine Learning" OR* TI ML *OR* AB ML *OR* TI *"Natural Language Processing" OR AB*	897

	“Natural Language Processing” OR TI NLP OR AB NLP OR TI ChatGPT OR AB ChatGPT OR TI LLM OR AB LLM OR TI “Deep Learning” OR AB “Deep Learning” OR TI “Transformer Model” OR AB “Transformer Model” OR TI “Neural Network*” OR AB “Neural Network*” OR TI “Text Mining” OR AB “Text Mining” OR TI “Automated Text Analysis” OR AB “Automated Text Analysis” OR TI “Conversational AI” OR AB “Conversational AI” OR TI “Generative Model*” OR AB “Generative Model*” OR TI “Foundation Model*” OR AB “Foundation Model*” OR TI “Prompt Engineering” OR AB “Prompt Engineering”)) AND ((TI “Thematic Analysis” OR AB “Thematic Analysis” OR TI “Content Analysis” OR AB “Content Analysis” OR TI “Qualitative Research” OR AB “Qualitative Research” OR TI “Interpretative Phenomenological Analysis” OR AB “Interpretative Phenomenological Analysis” OR TI “Discourse Analysis” OR AB “Discourse Analysis” OR TI Ethnography OR AB Ethnography OR TI “Participant Observation” OR AB “Participant Observation” OR TI “Reflexive Thematic Analysis” OR AB “Reflexive Thematic Analysis” OR TI Autoethnography OR AB Autoethnography OR TI “Member Checking” OR AB “Member Checking” OR TI “Data Saturation” OR AB “Data Saturation” OR TI “Inductive Analysis” OR AB “Inductive Analysis” OR TI “Deductive	

	Analysis" OR AB *"Deductive Analysis" OR* DE *"Empirical Research" OR* DE *"Focus Groups" OR* DE *"Interviews" OR* DE *"Narratives" OR* DE *"Observation")*	
